# Experiences of Individuals Who Were Physically Restrained in the Emergency Department

**DOI:** 10.1001/jamanetworkopen.2019.19381

**Published:** 2020-01-24

**Authors:** Ambrose H. Wong, Jessica M. Ray, Alana Rosenberg, Lauren Crispino, John Parker, Caitlin McVaney, Joanne D. Iennaco, Steven L. Bernstein, Anthony J. Pavlo

**Affiliations:** 1 Department of Emergency Medicine, Yale School of Medicine, New Haven, Connecticut; 2 Yale School of Public Health, New Haven, Connecticut; 3 Rowan University School of Osteopathic Medicine, Stratford, New Jersey; 4 Medical College of Georgia, Augusta; 5 Yale School of Nursing, Orange, Connecticut; 6 Department of Psychiatry, Yale School of Medicine, New Haven, Connecticut

## Abstract

**Importance:**

Individuals with behavioral disorders are increasingly presenting to the emergency department (ED), and associated episodes of agitation can cause significant safety threats to patients and the staff caring for them. Treatment includes the use of physical restraints, which may be associated with injuries and psychological trauma; to date, little is known regarding the perceptions of the use of physical restraint among individuals who experienced it in the ED.

**Objective:**

To characterize how individuals experience episodes of physical restraint during their ED visits.

**Design, Setting, and Participants:**

In this qualitative study, semistructured, 1-on-1, in-depth interviews were conducted with 25 adults (ie, aged 18 years or older) with a diverse range of chief concerns and socioeconomic backgrounds who had a physical restraint order associated with an ED visit. Eligible visits included those presenting to 2 EDs in an urban Northeast city between March 2016 and February 2018. Data analysis occurred between July 2017 and June 2018.

**Main Outcomes and Measures:**

Basic participant demographic information, self-reported responses to the MacArthur Perceived Coercion Scale, and experiences of physical restraint in the ED.

**Results:**

Data saturation was reached with 25 interviews (17 [68%] men; 18 [72%] white; 19 [76%] non-Hispanic). The time between the patient’s last restraint and the interview ranged from less than 2 weeks to more than 6 months. Of those interviewed, 22 (88%) reported a combination of mental illness and/or substance use as contributing to their restraint experience. Most patients (20 [80%]) said that they felt coerced to present to the ED. Three primary themes were identified from interviews, as follows: (1) harmful experiences of restraint use and care provision, (2) diverse and complex personal contexts affecting visits to the ED, and (3) challenges in resolving their restraint experiences, leading to negative consequences on well-being.

**Conclusions and Relevance:**

In this qualitative study, participants described a desire for compassion and therapeutic engagement, even after they experienced coercion and physical restraint during their visits that created lasting negative consequences. Future work may need to consider more patient-centered approaches that minimize harm.

## Key Points

**Question:**

How do individuals interpret experiences of physical restraint in the emergency department?

**Findings:**

This qualitative study of 25 patients who were physically restrained in the emergency department found the 3 following major themes: harmful experiences of restraint use and care provision, diverse and complex personal contexts affecting visits to the emergency department, and challenges in resolving their restraint experiences, leading to negative consequences on well-being.

**Meaning:**

Results of this study suggest that the participants in this study desired compassion and therapeutic engagement during physical restraint, warranting further attention to patient-centered approaches and coercion-reduction techniques that fit with the needs of emergency care.

## Teaser

This qualitative study characterizes how individuals interpret experiences of physical restraint in the emergency department.

## Introduction

Visits to the emergency department (ED) related to behavioral disorders are rapidly increasing in the United States,^1^ with 1.7 million episodes of associated patient agitation occurring annually in emergency settings.^2^ A recent study performed at a large urban county health care facility^3^ estimated that agitation was associated with 2.6% of all ED visits. Unfortunately, treatment of these agitation episodes may lead to potential harm among patients. When deescalation attempts fail, ED staff commonly use physical restraints, which are associated with lasting physical injuries and cardiac arrest.^4,5,6^ Efforts to reduce threats to patient safety during agitation management have led to calls for early detection and intervention to prevent escalating agitation and to minimize the use of restraints.^7^

However, use of deescalation is often challenging in the emergency setting, where the acuity of agitation may be higher and the nature of patient presentations is more varied.^8^ Behavioral techniques rely on building a strong rapport and therapeutic relationship with the patient, but ED staff have reported that their insight into patients’ perspectives during agitation events is very limited.^9^ This is compounded by the fact that agitated patients may often experience *coercion*, defined as actions performed on a patient that cause loss of self-determination, ranging from involuntary detention or seclusion to use of forcible treatment with restraints.^10^ A recent observational study^11^ found that 61% of agitated patients felt coerced by police or prehospital staff before ED arrival. Experiences of coercion may limit the ability of ED clinical staff to engage in a dialogue and create a therapeutic bond.^12^

Unfortunately, studies that are drawn directly from the perceptions and experiences of patients who experienced agitation episodes in the ED are rare. Having a better understanding of patient wishes during and experiences of ED agitation care may assist staff in practicing the use of noncoercive techniques at the bedside. This study aimed to explore the experiences, backgrounds, and health care system interactions of individuals who were physically restrained during an ED visit. In giving these patients a voice, we hope to inform future research, policy, and clinical practice for the use of restraints and management of agitation in the ED.

## Methods

### Study Design

This was a qualitative study using a grounded theory approach, seeking to describe how individuals experience being physically restrained in the ED.^13^ The research team consisted of an interprofessional group with expertise in clinical psychology, public health, psychiatry, systems engineering, and emergency medicine to provide a broad spectrum of perspectives, given the wide etiologies of agitation and reasons to use restraints in the emergency setting. Our study protocol was approval by the Yale University institutional review board. We followed the 32-item Consolidated Criteria for Reporting Qualitative Research (COREQ) reporting guideline.^14^

### Participants and Setting

Our study sites consisted of a tertiary care academic referral center and a community-based teaching hospital with average annual adult ED volumes of 99 000 and 62 000 visits, respectively. Both institutions belong to a large regional health care network in the Northeast United States. Our previous work^15^ identified approximately 1300 unique adult visits per year to these EDs that were associated with a physical restraint. Eligible individuals were adult ED patients who had been restrained during a visit to either hospital site. We identified these patients either through a convenience sample of visits that contained an ED restraint order between March 2016 and February 2018 or during a prospective cohort study^11^ of agitation events that occurred in either study site ED between June 2017 and August 2017. Participants received $50 in compensation for completion of a survey and interview. We obtained verbal informed consent from our participants at the beginning of each session.

### Study Protocol and Data Collection

To represent the breadth of classes and backgrounds of patients who may be restrained in the ED, we performed purposive sampling in an iterative fashion throughout the data collection period. We identified the demographic and clinical characteristics of patients restrained in the ED at our study sites over a previous 3-year period, as follows: a median age of 46 years, 68.3% men, 73.5% white individuals, and 22.1% homeless individuals.^15^ We then used this data to recruit a sample with a similar representation of sex, race/ethnicity, reason for ED visits, and other relevant demographic characteristics. Experts have identified that patients’ perceptions of coercion may differ depending on the interval between the restraint episode and data collection.^16^ Thus, we also sampled for a range of periods between the date of last ED restraint episode and the date of interview. We contacted a total of 79 eligible individuals by telephone, with 45 (57%) either having telephone numbers that no longer functioned or not responding to our request to call back. A total of 4 individuals (5%) declined to participate.

We developed interview questions through a synthesis of previous literature on patients’ experiences of coercion^16,17^ and triangulation with perceived patient perspectives from qualitative data of ED staff regarding the use of restraints.^18^ The interview guide explored participant experiences in the ED, associated health conditions, reflections on restraint episodes, and consequences on health outlook and interactions with health care. One member of the research team (A.R.), who was trained in qualitative data collection, conducted the in-person interviews. A second team member (J.P. or C.M.) made field notes during the sessions. Discussions lasted between 40 and 60 minutes and were audio recorded on digital equipment, professionally transcribed verbatim, deidentified, and entered into qualitative data management software (Dedoose version 8.2.27; SocioCultural Research Consultants).

At the end of each interview, we conducted a brief survey to collect anonymized sociodemographic information and responses to the MacArthur Perceived Coercion Scale (MPCS-5),^19^ a brief 5-question instrument used in the psychiatric literature to measure perceived coercion and represent patients’ sense of empowerment, choice, and personal influence associated with health care visits.

### Statistical Analysis

A 5-member coding team (A.H.W., A.R., C.M., A.J.P., and J.P.) used Dedoose for thematic analysis and organization of the qualitative data. The coding team created and refined the code book through masked open coding of initial transcripts, identifying additional codes through iterative rounds of group discussion, and integrating existing codes. After all transcripts were analyzed, the team summarized codes into major themes and subthemes. We conducted descriptive statistics for the sociodemographic and MPCS-5 data using SPSS statistical software version 21.0 (IBM Corp).

## Results

### Characteristics of Study Participants

We achieved data saturation after interviews with 25 patients (17 [68%] men) who had been physically restrained during an ED visit. Those interviewed were predominantly white (18 [72%]) and non-Hispanic (19 [76%]) individuals. The interval between the patient’s last restraint and the interview ranged from less than 2 weeks to more than 6 months, with 10 patients (40%) being interviewed between 2 weeks and 1 month after restraint. Characteristics and patient factors, including homelessness and rates of alcohol use, drug use, and mental illness, are detailed in Table 1. A total of 22 participants (88%) reported a combination of mental illness and/or substance use as contributing to their restraint experience. When asked questions from the MPCS-5,^19^ 17 (68%) responded false to the statement, “I chose to come into the hospital,” while 20 (80%) responded false to the statement, “I had more influence than anyone else on whether I came into the hospital,” indicating that most patients viewed entry to the ED as coercive (Table 2).

**Table 1.** Patient Characteristics and Attributes Associated With the Use of Restraints

Characteristic or Factor	No. (%) (N = 25)
Sex	
Men	17 (68)
Women	8 (32)
Race	
White	18 (72)
Black	7 (28)
Ethnicity	
Non-Hispanic	19 (76)
Hispanic	6 (24)
Age, y	
18-29	5 (20)
30-44	9 (36)
45-54	7 (28)
≥55	4 (16)
Interval between last restraint and date of interview	
<2 wk	4 (16)
2 wk to <1 mo	10 (40)
1 to <6 mo	5 (20)
>6 mo	6 (24)
Homelessness, current or prior	
Yes	8 (32)
No	17 (68)
Reported history of alcohol use, drug use, or mental illness	
Alcohol or drug use only	6 (24)
Mental illness only	4 (16)
Alcohol use and mental illness	3 (12)
Drug use and mental illness	5 (20)
Alcohol use, drug use, and mental illness	7 (28)
Factors associated with restraints	
Overall attitude or opinion toward restraints	
Negative	9 (36)
Mixed	10 (40)
Positive or self-blame	6 (24)
Reported reason for escalation of behavior	
Alcohol use	7 (28)
Drug use	3 (12)
Mental illness	6 (24)
Alcohol or drug use and mental illness	6 (24)
Confrontation with personnel or staff	3 (12)

**Table 2.** Patient Responses to MacArthur Perceived Coercion Scale^19^

Question	No (%)
“I felt free to do what I wanted about coming into the hospital”	
True	8 (32)
False	17 (68)
“I chose to come into the hospital”	
True	8 (32)
False	17 (68)
“It was my idea to come into the hospital”	
True	7 (28)
False	18 (72)
“I had a lot of control over whether I went into the hospital”	
True	5 (20)
False	20 (80)
“I had more influence than anyone else on whether I came into the hospital”	
True	5 (20)
False	20 (80)
“How did being brought to the hospital make you feel?”	
Angry	15 (60)
Sad	16 (64)
Pleased	4 (16)
Relieved	8 (32)
Confused	11 (44)
Frightened	12 (48)

### Qualitative Results

Qualitative analysis of these interviews identified the 3 following primary themes characterizing the experiences of individuals restrained in the ED: (1) harmful experiences of restraint use and care provision, (2) diverse and complex personal contexts affecting visits to the ED, and (3) challenges in resolving their restraint experiences, leading to negative consequences on well-being. Table 3 provides a summary of each of these themes as well as their subthemes, concepts, and definitions. Patient quotes illustrating subthemes and concepts are presented in Table 4. These tables are designed to present a comprehensive review of our results, while the following sections highlight key findings from each theme.

**Table 3.** Taxonomy of Concepts Describing Experiences of Individuals Who Have Been Restrained in the ED

Theme	Subtheme	Concept	Definition
Harmful experiences of restraint use and care provision	Lack of patient-centeredness	Dehumanization during restraint process	Loss of personal dignity through removal of clothing or property and lack of privacy in public places or hallway spaces
		Loss of self-determination and freedom	Patients are coerced against their wishes during their visit, often for basic human needs like bathroom use, food and water, and allowances for sitting up, getting off the stretcher, or pacing in the room
		Mistreatment or suboptimal treatment	Pain and physical discomfort from restraints, needles, and excess use of violence or force from personnel as well as lack of proper attention to the patient’s original reason for coming to the ED
	Emotional responses and consequences	Abandonment and isolation	A sense of being intentionally ignored or placed away from clinicians, especially after being restrained
		Confusion and uncertainty	Reasons for being restrained or treated in a certain fashion are unclear or contradictory, leading to difficulty in interpreting their visit experiences
		Frustration and anger or outrage	Personal agency and free will are lost, especially against perceived mistreatment, abuse, or overuse of force
		Worry, anxiety, and fear	Feelings of panic and stress from being disempowered for their own freedom or self-determination
Diverse and complex personal contexts affecting visits to ED	Physical and mental health	Consequences of health deterioration, disabilities, or chronic conditions	Declining health or active medical issues hinder and affect quality of life
		Difficult engagement with health network	Feelings of abandonment and futility with a health care system that is perceived to be unresponsive or uncaring, often despite frequent encounters or visits
		Responsibility and ongoing struggle for personal health and well-being	Ownership over their health and taking action to make it better and developing resilience and/or mechanisms to cope with difficulties
	Drug and alcohol use	Overdose and intoxication	Episodes of drug or alcohol use that lead to loss of control or personal safety, often leading to entry in ED
		Shame and struggle against drugs and/or alcohol	Path to recovery and associated regret and embarrassment for struggles to combat substance use disorders
		Treatment and referral	Participation in rehabilitation and detoxification programs, with mixed results among individuals
	Social determinants and backgrounds	So-called downward spiral	Significant psychosocial stressors that deteriorate or debilitate life path, leading to homelessness, lack of stable income and/or employment, legal trouble, and/or incarceration
		Occupational and social backgrounds	Additional perspectives about their restraint or visit experiences because of prior job training or social experiences (eg, nursing, security, or law enforcement)
		Physical and sexual abuse	Physical and psychological trauma in their lives heighten and exacerbate use of force and restraint against them
Challenges in resolving their restraint experiences, leading to negative consequences on well-being	Interpretations and opinions on restraint use	Respect for personal freedom and rights as top priority	Expression of their desire to be left alone or to be allowed to make choices regarding their disposition or treatment, even if it may threaten their own safety
		Self-reflection on their own role in restraint use	Admission of their own behavior contributing or leading to use of restraints, leading to a spectrum of feelings including self-blame, self-forgiveness, and seeking closure to their ED experiences
		Insight into use of coercion against their wishes	Acceptance and/or understanding that coercive measures need to be used or may be necessary in certain instances to provide safety for staff or themselves
	Consequences on outlook and future actions	Distrust of health care system and professionals	Fracturing of engagement or seeking help for physical and mental health because of restraint experiences
		Inevitability and futility of ED visits	Treatment plans or pathways through ED go according to decisions outside of their control, and patients accept or realize that it will happen regardless of their actions, wishes, or requests
		Personal lives affected as a result of ED visit	Long-term negative consequences and harm on psychological, social, and physical well-being

Abbreviation: ED, emergency department.

**Table 4.** Key Quotes Demonstrating Themes

Theme	Subtheme	Quote
Harmful experiences of restraint use and care provision	Lack of patient-centeredness	“I was in the hallway the whole time. Nobody says or does nothing. The longer that you restrain me personally, the more I’m going to be stressed out. All I did was ask them to just release my arms. I don’t care about being restrained. Leave them on my legs, it doesn’t bother me on my legs. Let me wipe my face or itch my nose or whatever. There’s no need to treat me like an animal. It’s uncalled for. Honestly.”
		“I’m in here probably for substance abuse and I’m homeless, so they look at me like, ‘You are the least of our worries right now. You are not important enough to even talk to, right now. We’ll get to you when we have time,’ or something. There could be nothing really going on in the hospital but seeing how I’m in here for whatever reason, I’m the very last person to be seen because I’m homeless, or whatever the case may be.”
	Emotional responses and consequences	“I was intoxicated, so I was scared and I guess, I was too wild, and they had to stick me with the needle in my bottom part. The next minute, you know, I woke up with tubes down my nose, IV in my arm, and I was scared, I didn’t know what happened, and the doctor just tells me to calm down. Obviously, there was a reason why they put the tube in my nose, maybe I wasn’t breathing right or anything like that. I felt like I was knocked out for hours. They just told me that they had to restrain me because I was too wild at the moment in time when I was intoxicated. The only thing I really wanted was my credit card, I lost it at the club. I guess they was scared, so they had to put me to sleep. It was scary for me. I had no control over my body.”
		“You have no clue what is going on. It’s really confusing. When you are on a medication they inject into you, you don’t know why you are acting like this. I was really lost. I didn’t know anything and then when they restrain you they ignore you. If you took 2 minutes out and said, ‘Listen, we put you this way for this reason. This is how long it’s going to take. When this period is done we will release you or take you out of restraints.’ It’s just that I was automatically restrained and nobody tells you why.”
Diverse and complex personal contexts affecting visits to the emergency department	Physical and mental health	“I call it a curse because you can go about your business, whatever you’ve got going on, and then all of a sudden for whatever odd reason, chemical imbalance, maybe. You could just go manic and you are not yourself. Then you have to get medicated, hospitalized, and then come down from the overmedication until you feel like yourself again. After I had my son, I started taking medication to control it, but then I’m manic again, so it was unpreventable even then. I don’t know. It’s my brain is different from other people.”
		“I started pushing on the damn boil, getting all the pus out, cleaning it up with Dakin’s [antiseptic] that I got after surgery. I do everything in my power not to go the hospital, I really wait to the last minute when I know it’s so infected, the skin is hot, I have a fever. My father yells at me all the time. He’s like, ‘You’re going to die,‘ because it’s true. But you don’t understand what I have to go through every time I’m there, they poke and prod at me and treat me like dirt in the emergency department because they think I’m there to get pain medications or whatever.”
	Drug and alcohol use	“I found out I had HIV. That was one of the reasons why I started smoking dust, because of the stress and the denial. I started smoking dust and don’t think about it. Then I realized I can’t do that because it affects me and it make it worse. I realized I’m only hurting myself and I have a daughter. She’s 15 now. I said I have to be here for my daughter as long as I can be. I stopped doing that. I started taking the meds that they gave me. The smoking is where we got to the restraint parts, when I would lose control and get dragged to the hospital.”
		“Well, it’s extensive. One time I was abusing cocaine, and I went in there and my heart rate was like 220. They were doing everything they could do to help stabilize me. That wasn’t that long ago, not long enough ago really. I do these things to myself, and then I don’t know what my expectation is going into it really. I just know that at some point I kind of cross a line with the alcohol too. Then I do things that are not really characteristic of me on a level-headed basis.”
	Social determinants and backgrounds	“I know [name of study institution] is a very good hospital, university. But I believe, because in my case I’ve been a school bus driver, if you have any personal problems you have to leave it at home because the kids are tough, the parents are tough, so you have to be playing, like it or not, as a professional. I believe this nurse also has to act like a professional. Because when you have the kind of career that she took, okay, it’s good money, but you have to be polite. You have to understand the patient, treat them with love. At least a little bit of love. Because you don’t even know what the patient might be going through.”
		“My parents often drank and my dad would beat my mother. My older brothers would hide us or shove us in a room and lock the door. We would hear my mom screaming and not be able to do anything about it. I was also sexually abused as a child. The little that I can remember, because I was very young, there was some type of restraint, so I think that’s the worst thing when they hold me down and restrain me like that.”
Challenges in resolving their restraint experiences, leading to negative consequences on well-being	Interpretations and opinions on restraint use	“It’s because I don’t want to be in the hospital. They tried to ignore me and end up restraining me because they tried to seclude my anger. No, I’m p-ssed off. I don’t care if I hurt myself or anyone else, just let me… out.”
		“I was married to a doc. When I met her—I was investigating a homicide when I met her, and she was doing a residency, rotating through the ER when I first met her. She was at Mass General. I’ve been around docs all my life. Sh-t happens. That’s just it. I’m at the point now I’m embarrassed that that did happen, but I don’t beat myself up for it anymore. There’s nothing really to be ashamed of. Can’t take the bullet back. Once it’s out of the gun, it’s gone. It’s done, it’s done. It’s over. Hopefully, it won’t happen again.”
	Consequences on outlook and future actions	“I will live with this broken finger, they did that to me when they held me down like that. You will not break my finger again. I remember that pain and that pain didn’t go away. I was in pain for a long, long time. I think that from the bone being jammed down, it’s affecting this finger. This finger, when I bend it, it gets stuck. I have to physically push it back up because it hurts. I don’t know. If the security can get away with breaking somebody’s finger and nothing be done about that, then how can they call it a hospital?”
		“The social worker called DCF. They came 3 times already. I didn’t drink. I peed in the cup at mental health. I’ve seen my counselor. All this stuff happened after I left the emergency room. I didn’t even know my daughter got a phone call. ‘DCF is comin’.”
		“Once I’m in that emergency, oh, there ain’t no way I’m leaving there and going home. That’s why I already know. When my sisters call the paramedics. Oh it’s going to be a while. I just go kiss my son, see you later. The experience in the emergency room, it’s traumatic as hell and it makes me feel like a piece of sh-t.”

Abbreviation: DCF, Department of Children and Families.

#### Harmful Experiences of Restraint Use and Care Provision

In recalling the experience of restraint, patients described a loss of freedom and personal dignity associated with dehumanization, loss of self-determination, and even mistreatment. One patient said, “You took all my clothes off, you had me laying on the bed strapped down with no clothes, no cover, no nothing. My privates are wide open, people just walking by, and you won’t give me no clothes or shut the curtain.” The patient experience of restraint ranged from descriptions of being treated like an animal to being handled roughly both physically and verbally by staff, even including displays of overt antagonism and profanities. One participant reported, “The lady who told me to shut up, one of the nurses, she pushed my face in like that. I say, ‘I can’t breathe; you’re hurting my neck. Let me go. You people are hateful the way you treat me.’ After I was tied up, she just gave me the finger. The policeman who was nice to me, he just looked at her like he knows she’s doing something wrong, but they don’t say anything.”

Emotional responses to the restraint experience included confusion, frustration, worry, and a sense of isolation. This may reflect the fact that most interviewed patients (17 [68%]) did not freely choose to enter the ED in which they were restrained. A frequent analogy when describing the restraint experience was that of being in prison. “I felt like nobody really cared. I felt like I was in prison, in the bed. I’ve never been to jail before, so my first experience of it was scary for me. I’d never been restrained before, I never had anyone hold me from my rights, you know? I cried, you know, I felt like I was alone in the bed with the straps on my wrists, my ankles.”

Patients described the experience of not being heard or acknowledged by anyone around them, leading them to conclude that ED staff did not care about them or were even actively shunning them. “The people that take care of you on the other floors are considerate and care and talk to you, but the people in the ED ignore you. They don’t care about you. They walk by you a hundred times. You can try to ask them a question every time they walk by and nobody will say anything, they’ll just keep walking right by you as if they can’t hear you.”

#### Diverse and Complex Personal Contexts Affecting Visits to the ED

A number of medical and social factors were found to shape the individual contexts preceding and following restraint experiences. Participants described facing challenges and ongoing struggles with drug and alcohol use, mental health, or complex and chronic medical problems that were difficult to manage and significantly affected their daily lives. In addition, the perception of an unresponsive, unavailable, and inconsistent health care system led some patients to experience difficulty engaging with the health care system as a whole, despite sometimes serious medical conditions. One patient explained, “The beginning of this year, I started doing dope because I been suffering through this pain for such a long time. It started out as a small leg issue and they messed up big time when they took me to surgery. Now it’s affected different areas of my body. It’s not just the physical part of it. I can’t really do what I want to do like play basketball or martial arts—the things that I would really like to do for my life. The doctor that initially did the surgery wasn’t qualified and caused all these problems, and then I kept being sent to a different doctor after another until I gave up.”

Social determinants, including psychosocial stressors, occupational and social backgrounds, and histories of being survivors of abuse, further shaped their visits to the ED and their interactions with staff during the restraint experience. In relating experiences of homelessness to an ED visit, a participant stated, “It just seemed like, almost every month I was ending up [in the ED]. My ankle was so bad I couldn’t get around, I couldn’t eat. They had to admit me because I couldn’t walk or really do anything. It was more like my social situation was really intense than it was a medical thing, but what else could I do?”

#### Challenges in Resolving Their Restraint Experiences, Leading to Negative Consequences on Well-being

Interview analysis revealed that restraint experiences affected not only proximal relationships within a given visit but had lasting consequences on a patient’s relationship with the health care system. Reflecting on their experiences produced a range of reactions among the participants regarding the use of restraints in the ED, from a desire for personal freedom to self-reflection and understanding. On one end of this spectrum patients described the desire to maintain free will, even in the setting of attempting to self-harm. These sentiments are often because of apprehension and skepticism of the underlying intentions of health care professionals and society at large. “Honestly, I think if someone’s going to kill themselves, it’s their choice. They should be able to kill themselves. People should be left free to do what they want to do, even if it means it hurts them. The folks in the hospital, they don’t really care if you do it or not.” In contrast, other patients’ reflections describe an understanding of caregiver intent, usually when they view the role of their own state more strongly at the time of restraint. For example, a participant said, “The staff have got my best interest at heart. They’re trying to do the best that they can, but if it happens once, twice, that’s a different story. When it happens 6, 7 times, it’s like—they get sick of it. I don’t blame them. They have other things that they could be dealing with and helping other people.”

Patients described developing distrust and avoidance of health care interactions, perceiving the outcome of ED visits as inevitable, and leaving the ED with lasting physical and psychological consequences from their restraint experience. Repeated exposure to similar experiences led patients to a feeling of inevitability or futility in the path of their ED experience. One participant explained, “I already experienced so many times when they go right to the straps, to me it’s a ritual. It’s just what it is. There’s nothing I can do about it.” For some patients, these experiences led to a distrust of and disengagement from the health care system, especially when they were associated with adverse effects because of the use of restraints and sedatives. For example, a participant said, “I think they need to be a little more conscious about what they give people because obviously, they did something incorrect that I ended up in the ICU [intensive care unit]. I think they need to be a little more careful with the dosage of that strong stuff that they give you, I couldn’t even hold my head up, I was drooling. It’s terrible, and I still have flashbacks about that. I don’t want to go back to that again, ever.”

Other lasting consequences of restraint use and ED visits included the exacerbation of existing psychiatric conditions. One participant reported, “After all the times I’ve been restrained in the emergency room, it makes my PTSD and anxiety worse. My provider increases my anxiety medication for a few days until I can adjust to being back outside and get it out of my mind.”

## Discussion

Using a qualitative approach, we found that those who were physically restrained during their visits to the ED represented a marginalized population that desired compassion from and therapeutic engagement with the staff caring for them. These individuals frequently presented at times of crisis requesting or requiring emergency care. Our findings indicated that, of those interviewed, most did not enter the ED willingly, as indicated during their interviews and in their responses to the MPCS-5.^19^ In addition, our participants felt violated and dehumanized, with a lack of connection to and understanding of the clinical decisions, process, and events leading up to the use of restraints. In fact, some perceived mistreatment and overt physical violence against them, leading to a range of negative responses both immediately and after discharge. These experiences were influenced by homelessness, drug and/or alcohol use, physical and mental illness, and inadequate or harmful system responses to these concerns, resulting in the repetition of agitation events during times of intense stress. Finally, their experiences of being restrained in the ED led to challenges in interpreting future outlook and implications, with many describing health care avoidance, mistrust of the public health system, and lasting consequences that they continued to struggle with as a result of being restrained in the ED. These new insights into the experiences and contexts of these patients may help inform future investigations and changes in clinical practice for the use of restraints and management of agitation in acute care settings.

Understanding and addressing the needs of individuals with drug and/or alcohol use disorder and mental illness is a pressing issue. However, there are limited primary sources that describe experiences of those patients during crisis and decompensation of their disease, leading to coercion and visits to the ED. Within similar contexts in psychiatry, patients with mental illness who experienced coercion during their inpatient psychiatric admissions reported negative feelings of helplessness, fear, and rage, with increased sentiments of isolation and despair among the cohort that was physically restrained.^20,21^ In contrast, a previous study of ED patients who received sedatives in a large metropolitan Australian hospital^17^ found a significantly different set of experiences among their participants, with many describing positive sentiments of respect, dignity, and trusting relationships with staff members. Unfortunately, our interviewees described overall negative experiences and emotional consequences, with some even perceiving overt antagonistic and unprofessional behavior from staff members during their restraint process and associated ED visit. These contrasting findings may be because of disparate geographic and clinical practice conditions, pathways to the ED, or differences in focus between sedative use vs restraint use.

Recent studies examining the experiences of ED health workers during the management of agitation have begun to shed light on potential reasons for our patients’ descriptions of their restraint experiences. Staff members^18^ reported that decompensation of mental illness and disinhibition from alcohol and/or drug use manifested as loud and boisterous behavior in agitated patients. This described behavior may have resulted in the need for a level of attention that was often unable to be met in the busy ED environment and led to feelings of neglect and abandonment in our participants. In addition, our previous work found that staff sentiments of empathy and compassion for this patient population were sometimes overshadowed by fear and frustration because of verbal and physical assaults they encountered from the same patients they desired to care for.^9^ These sentiments may have manifested as episodes of mistreatment or displays of aggression that our participants described during their experiences of being restrained. Thus, systems that address potential staff harm and patient safety together as part of the same problem would enable health care professionals at the bedside to make conscious, balanced, and evidence-based decisions to use physical restraints only as a last resort and minimize associated lasting negative consequences on the patients under their care.

### Limitations

Our study contains several key limitations. First, a number of patients were identified as eligible who either did not respond to the study request (57% of those contacted) or declined to participate (5%). It is possible that these patients had different experiences than those who chose to participate in our study. In addition, it is possible that the time between restraint and our interview could have affected the recollection of the restraint experience. Memory processing and memory decay over time could affect the recall of the event as well as the emotions surrounding the event.^22^ In an attempt to control for this, we purposely sampled patients with a spectrum of intervals between restraint experience and the interview.

## Conclusions

The participants in this study represented the most marginalized and disadvantaged population that presents to the ED. They overwhelmingly described a desire for dignity, respect, compassion, therapeutic engagement, and attempts by staff to explain actions performed on them, even if the therapeutic relationship has been fractured as a result of coercion and physical restraint during agitation events. Most participants did not present to the ED willingly and already felt threatened before arrival. Without psychological support from staff members during these restraint events, patients may begin a spiral of negative emotions with subsequent ED visits and instinctively escalate with short-term agitation and aggression as a protective shield based on prior experiences. Instead of providing care, we may inadvertently cause more harm to these individuals. Our findings suggest that patient-centered approaches to agitation management may be helpful to foster a therapeutic alliance, which may include documentation of specific behavioral health care plans^23^ tailored for patients and described in their electronic health records or case-management programs^24,25^ that link with outpatient and social services for patients with complex mental health conditions and histories of violent behavior. Future work may include partnering with patient advocates or community members to develop better behavioral care processes and adapt agitation-reduction techniques that reduce coercion to fit with the needs of emergency care.

## References

[1] Capp R, Hardy R, Lindrooth R, Wiler J National trends in emergency department visits by adults with mental health disorders. J Emerg Med. 2016;51(2):-. 27614303 10.1016/j.jemermed.2016.05.002

[2] Holloman GH Jr, Zeller SL Overview of Project BETA: Best practices in Evaluation and Treatment of Agitation. West J Emerg Med. 2012;13(1):1-2. doi:10.5811/westjem.2011.9.6865 22461914 PMC3298232

[3] Miner JR, Klein LR, Cole JB, Driver BE, Moore JC, Ho JD The characteristics and prevalence of agitation in an urban county emergency department. Ann Emerg Med. 2018;72(4):361-370. doi:10.1016/j.annemergmed.2018.06.001 30031556

[4] Mohr WK, Petti TA, Mohr BD Adverse effects associated with physical restraint. Can J Psychiatry. 2003;48(5):330-337. doi:10.1177/070674370304800509 12866339

[5] Zun LS A prospective study of the complication rate of use of patient restraint in the emergency department. J Emerg Med. 2003;24(2):119-124. doi:10.1016/S0736-4679(02)00738-2 12609639

[6] Karger B, Fracasso T, Pfeiffer H Fatalities related to medical restraint devices: asphyxia is a common finding. Forensic Sci Int. 2008;178(2-3):178-184. doi:10.1016/j.forsciint.2008.03.016 18455336

[7] Knox DK, Holloman GH Jr Use and avoidance of seclusion and restraint: consensus statement of the American Association for Emergency Psychiatry Project BETA Seclusion and Restraint Workgroup. West J Emerg Med. 2012;13(1):35-40. doi:10.5811/westjem.2011.9.6867 22461919 PMC3298214

[8] Downey LV, Zun LS, Gonzales SJ Frequency of alternative to restraints and seclusion and uses of agitation reduction techniques in the emergency department. Gen Hosp Psychiatry. 2007;29(6):470-474. doi:10.1016/j.genhosppsych.2007.07.006 18022038

[9] Wong AH, Combellick J, Wispelwey BA, Squires A, Gang M The patient care paradox: an interprofessional qualitative study of agitated patient care in the emergency department. Acad Emerg Med. 2017;24(2):226-235. doi:10.1111/acem.13117 27743423

[10] Sashidharan SP, Mezzina R, Puras D Reducing coercion in mental healthcare. Epidemiol Psychiatr Sci. 2019;28(6):605-612. doi:10.1017/S2045796019000350 31284895 PMC7032511

[11] Wong AH, Crispino L, Parker J, Use of sedatives and restraints for treatment of agitation in the emergency department. Am J Emerg Med. 2019;37(7):1376-1379. 30598374 10.1016/j.ajem.2018.12.027

[12] Harrison N, Mordell S, Roesch R, Watt K Patients with mental health issues in the emergency department: the relationship between coercion and perceptions of being helped, psychologically hurt, and physically harmed. Int J Forensic Ment Health. 2015;14(3):161-171. doi:10.1080/14999013.2015.1073195

[13] Curry LA, Nembhard IM, Bradley EH Qualitative and mixed methods provide unique contributions to outcomes research. Circulation. 2009;119(10):1442-1452. doi:10.1161/CIRCULATIONAHA.107.742775 19289649

[14] Tong A, Sainsbury P, Craig J Consolidated criteria for reporting qualitative research (COREQ): a 32-item checklist for interviews and focus groups. Int J Qual Health Care. 2007;19(6):349-357. doi:10.1093/intqhc/mzm042 17872937

[15] Wong AH, Taylor RA, Ray JM, Bernstein SL Physical restraint use in adult patients presenting to a general emergency department. Ann Emerg Med. 2019;73(2):183-192. doi:10.1016/j.annemergmed.2018.06.020 30119940

[16] Soininen P, Putkonen H, Joffe G, Korkeila J, Välimäki M Methodological and ethical challenges in studying patients’ perceptions of coercion: a systematic mixed studies review. BMC Psychiatry. 2014;14:162. doi:10.1186/1471-244X-14-162 24894162 PMC4051960

[17] Yap CYL, Knott JC, Kong DCM, Gerdtz M, Stewart K, Taylor DM Don’t label me: a qualitative study of patients’ perceptions and experiences of sedation during behavioral emergencies in the emergency department. Acad Emerg Med. 2017;24(8):957-967. doi:10.1111/acem.13218 28500785

[18] Wong AH, Ruppel H, Crispino LJ, Rosenberg A, Iennaco JD, Vaca FE Deriving a framework for a systems approach to agitated patient care in the emergency department. Jt Comm J Qual Patient Saf. 2018;44(5):279-292. doi:10.1016/j.jcjq.2017.11.011 29759261

[19] Gardner W, Hoge SK, Bennett N, Two scales for measuring patients’ perceptions for coercion during mental hospital admission. Behav Sci Law. 1993;11(3):307-321. doi:10.1002/bsl.2370110308 10150233

[20] Steinert T, Birk M, Flammer E, Bergk J Subjective distress after seclusion or mechanical restraint: one-year follow-up of a randomized controlled study. Psychiatr Serv. 2013;64(10):1012-1017. doi:10.1176/appi.ps.201200315 23771400

[21] Georgieva I, Mulder CL, Whittington R Evaluation of behavioral changes and subjective distress after exposure to coercive inpatient interventions. BMC Psychiatry. 2012;12:54. doi:10.1186/1471-244X-12-54 22647058 PMC3412723

[22] Strout TD Perspectives on the experience of being physically restrained: an integrative review of the qualitative literature. Int J Ment Health Nurs. 2010;19(6):416-427. doi:10.1111/j.1447-0349.2010.00694.x 21054728

[23] Gates D, Gillespie G, Smith C, Rode J, Kowalenko T, Smith B Using action research to plan a violence prevention program for emergency departments. J Emerg Nurs. 2011;37(1):32-39. doi:10.1016/j.jen.2009.09.013 21237365

[24] Pope D, Fernandes CM, Bouthillette F, Etherington J Frequent users of the emergency department: a program to improve care and reduce visits. CMAJ. 2000;162(7):1017-1020. 10763402 PMC1232308

[25] Kumar GS, Klein R Effectiveness of case management strategies in reducing emergency department visits in frequent user patient populations: a systematic review. J Emerg Med. 2013;44(3):717-729. doi:10.1016/j.jemermed.2012.08.035 23200765

